# Haptoglobin in Juvenile Idiopathic Arthritis

**DOI:** 10.1186/s12969-022-00777-5

**Published:** 2022-12-15

**Authors:** Lillemor Berntson, Jakob Palm, Fredrik Axling, Peter Zarelius, Per M. Hellström, Dominic-Luc Webb

**Affiliations:** 1 Present Address: Department of Women’s and Children’s Health, Uppsala University, Uppsala, 75185 Sweden; 2 Gastroenterology and Hepatology Unit, Department of Medical Sciences, Uppsala University, Uppsala, Sweden; 3 Department of Surgical Sciences, Uppsala University, Uppsala, Sweden

**Keywords:** Arthritis juvenile, Acute phase proteins, Haptoglobin, Biomarkers

## Abstract

**Background:**

Haptoglobin (Hp), a liver derived acute phase inflammatory protein (APP), has scarcely been studied in juvenile idiopathic arthritis (JIA). Hp can occur in blood as two isoforms (Hp1 and Hp2) in precursor and mature forms. Routine clinical chemistry immunoturbidimetry does not discern these forms. It is unknown how different forms relate to disease activity in JIA. Our aims were to determine allele frequency and plasma concentrations of different Hp forms at higher versus lower JIA disease activity and compare to other APPs.

**Methods:**

Plasma from JIA (*n* = 77) and healthy (*n* = 42) children were analyzed for apparent Hp allelic frequency and densitometric concentrations of alpha forms by Western blot (WB). Polymerase chain reaction (PCR) (buffy coat) was performed in a subset to estimate conformity with genetics. At higher versus lower juvenile arthritis disease activity score (JADAS27) (which includes erythrocyte sedimentation rate (ESR)), total mature Hp concentration from WB was compared and correlated against immunoturbidimetry and total protein, albumin, serum amyloid A (SAA) and C-reactive protein (CRP).

**Results:**

At 300-fold dilution needed to study mature forms in Western blot, precursors were undetectable. Hp2 contributed most signal in most samples. Hp allele frequency was similar in JIA and controls. Both mature forms, taken separately or by sum, declined following treatment, but remained above concentrations of healthy controls, even in a remission subset that achieved JADAS27 < 1. Densitometry correlated with immunoturbidimetry. Hp concentrations correlated with JADAS27, albumin (negatively), CRP and SAA with immunoturbidimetric method correlating strongest to JADAS27 (Spearman R ~ 0.6, *p* < 0.0001).

**Conclusion:**

Hp allele frequency in JIA is similar to the general population, indicating that children with JIA should have the same possibility as in healthy children to produce preHp2 (zonulin), thought to increase intestinal permeability. Circulating Hp concentrations largely parallel other APPs and ESR; none of these measures correlate very strongly to JADAS27 score but Hp can be measured from capillary sampling which is impossible with ESR.

**Supplementary Information:**

The online version contains supplementary material available at 10.1186/s12969-022-00777-5.

## Background

Juvenile idiopathic arthritis (JIA) is heterogeneous, comprising seven categories according to the International League of Associations for Rheumatism (ILAR) criteria [1]. It is mainly considered an autoimmune disease with apparent contributions from the adaptive and innate branches of the immune system [2, 3]. Alterations of the gastro-intestinal (GI) immune system, such as induced by early use of antibiotics or short period of breast-feeding after birth, have been shown to be risk factors for JIA [4, 5].

Gut permeability is normally tightly regulated. Hyper-permeability risks antigen exposure to the underlying tissues rich in immune cells. Increased gut permeability has been shown and discussed in JIA pathology [6, 7]. Non-steroidal anti-inflammatory drugs (NSAIDs), as well as disease modifying anti-rheumatic drugs (DMARDs), cause GI symptoms in JIA, but to what degree they influence permeability remains unknown [8]. The association between hyper-permeability and autoimmune diseases and the challenges in measuring permeability and interpreting results were recently published [9, 10]. The paracellular barrier in the mucosal epithelium is regulated by tight junctions [11].

Haptoglobin (Hp) is one of several circulating liver derived acute phase proteins (APPs) that change during inflammation, and a precursor of Hp is thought to increase intestinal permeability. Hp increases in response to interleukin 6 (IL-6). The IL-6 signal system is highly selective for activating liver derived APPs. We recently demonstrated strong correlations between CRP and serum amyloid A (SAA), but not with the majority of pro-inflammatory cytokines, such as IL-8 that rises in gut bacterial infections without concomitant rise in IL-6, CRP or SAA [12]. There is current interest in IL-6 blockade as treatment modality for JIA [13], prompting a need to better understand changes in IL-6 and liver derived APPs at different disease activity phases in JIA. IL-6 itself is not commonly measured in clinics for chronic inflammatory conditions due to its problematically short half-life in blood of a few hours. A precursor of mature Hp, pre-Hp2, also called zonulin, has been reported to increase gut permeability by action at the luminal surface by binding type 2 proteinase activated receptor at the small intestinal epithelium [14]. Hp is consistently found in human bile [15, 16], making contact between Hp and the luminal surface of the small intestinal mucosal epithelium plausible. Bile concentrations paralleling disease activity also seem possible, albeit bile cannot practically be accessed in JIA patients for direct investigation.

Hp is synthesized in the liver as a single polypeptide precursor that is cleaved into α- and β-chains that are then linked at disulfide bridges, yielding the mature forms. There are two different human Hp alleles, Hp1 and Hp2. These differ at α chains named α1 and α2. Thus, three phenotypes exist: Hp1-2, Hp1-1 or Hp2-2 [17]. Possession of at least one Hp2 allele is required to express pre-Hp2. Hp is measured in routine clinical chemistry by immunoturbidimetry. This method was not designed to differentiate pre-protein from mature forms or the two allelic isoforms. Earlier studies demonstrated increased Hp in JIA and suggested allele frequencies in JIA are comparable to healthy controls [18, 19]. It is unknown if these Hp forms change in JIA in relation to each other or in relation to other liver APPs or disease activity indexes, such as JADAS27.

The capacity for any APP to take part in JIA pathogenesis is not straightforward and somewhat counter-intuitive. ESR, which reflects fibrinogen, and CRP, which reflects IL-6, are among the oldest, most documented and most ordered inflammatory biomarkers in clinics, although the strength of their correlation in JIA is questionable. CRP is not a consistent and reliable indicator of JIA disease activity as measured by JADAS27 scoring, implying limited and inconsistent IL-6 elevation in circulation to drive increases in APPs. During inflammation, CRP can exhibit oscillatory concentration patterns and also comparatively short half-life. There are marked differences in half-lives of different APPs and any cyclic fluctuations would be unlikely to be in phase. Hence, it is possible for Hp (or other APPs) to correlate differently than CRP with ESR or JIA disease activity.

Primary aims were to determine Hp1 and Hp2 allele expression frequencies appearing in blood in JIA in comparison with healthy children and to measure plasma Hp1 and Hp2 concentrations in paired samples drawn at higher and lower disease activity. Secondary aims were to compare plasma Hp concentrations by Western blot to values obtained from routine clinical chemistry immunoturbidimetry and compare to other liver derived APPs.

## Methods

### Study subjects

Children aged 0–16 years of age (*n* = 77), diagnosed with JIA at the Unit of Pediatric Rheumatology, Uppsala University Hospital were included prospectively. Samples from another 42 healthy participants were collected pre-operatively from children admitted for minor surgery, for example orchidopexy or minor plastic surgery that were otherwise healthy and un-medicated. The list of exclusions was used by a pediatric nurse, trained to do this. Exclusion criteria were presence of any inflammatory disease, diabetes, any atopic disease with continuous medication or special diet because of intolerance, overweight (weight > 2SD according to standard curve).

### JIA classification and disease activity

At inclusion, JIA classification was done according to ILAR criteria [1]. Patients were included at onset of disease or if they were in a flare of the disease, representing higher individual disease activity state. Exclusion criteria in this study were any inflammatory co-morbidity, for example inflammatory bowel disease, or any hemolytic condition. Each patient also had follow-up at a comparatively lower disease activity state for that individual, and were reclassified according to ILAR criteria at follow-up. Disease activity at both visits was scored by JADAS27 (0–57) [20], comprising: (1) number of active joints (0–27); (2) patient global assessment visual analogue scale (VAS) (0–10 cm), assessed by a parent if the child was ≤ 9 years of age; (3) physician global assessment VAS (0–10 cm); (4) normalized erythrocyte sedimentation rate ((ESR in mm/h) − 20)/10) to a scale 0–10. Remission was defined as < 1.0.

### Blood samples

At each visit, blood was tapped into K_2_-EDTA tubes for plasma, which were sent directly to clinical chemistry (ESR, routine Hp by immunoturbidimetry, CRP and albumin) or processed and frozen for Western blot for Hp or cytokine panel for CRP and SAA. Prior to thawing plasma samples, a protease inhibitor cocktail was added, as detailed previously [21]. The cocktail consisted of KR-62,436 (DPP4 inhibitor, final 0.6 µM) and SIGMAFAST® protease inhibitor tablet (final 1X), both from Sigma-Aldrich Corp., St. Louis, MO, USA (Cat# K4264 and S8830). Buffy coat was drawn in a subset of samples for DNA analysis towards the end of this study, at which time ethics approval for this analysis was granted.

### Clinical chemistry

Plasma Hp concentrations by routine clinical chemistry used immunoturbidimetry at 604 nm absorbance on an Abbott Architect analyzer. The measurement interval for this assay was 0.1–2.7 g/L. Samples with concentrations above this were diluted and re-run. Reference interval (> 17 years of age) was 0.24–1.9 g/L. No reference data was available for those < 17 years of age. Healthy volunteers in this study recruited from the local population therefore provided reference data. CRP and SAA (liver derived positive APPs) were measured by multiplex ELISA (Meso Scale Diagnostics, MA, USA). The clinical chemistry unit assayed CRP (in some samples to bridge CRP values from the multiplex kit), albumin (negative APP) and ESR (to calculate JADAS27).

### Western blot

Herein, Hp1 refers to the shorter ancestral form with the corresponding peptide possessing one α subunit that gives rise to the 9 kDa subunit (α1). Hp2 refers to the longer allelic form possessing a duplication-fusion (effectively two tandem α subunits) that results in an ~ 18 kDa subunit (α2). Details of Western blot are in Additional file 1. Protein concentrations of plasma were measured by Bradford method. Plasma was diluted 1:300. Lanes were loaded with 1 µg protein. Each gel had one molecular weight marker lane and another for a reference sample (healthy plasma from heterozygote). Primary antibody was mouse monoclonal (clone E-9, IgG2b, Santa Cruz Biotech, Cat# sc-374,208, dilution 1:200). Densitometry was performed on both α-chains separately; ImageJ software was used [22]. Relative concentrations were calculated as ratio to the reference sample bands present on all membranes. The reference sample had the following concentrations: albumin (43 g/L), Hp (1.4 g/L by routine clinical chemistry immunoturbidimetry), protein (63 g/L). The sum of the α1 and α2 densitometries was used for “total Hp”. A sum equal to that of the reference sample was assigned a value of 1.0; a sum half of the reference was assigned a value of 0.5, etc.

### DNA analysis of hp alleles by PCR

Details of this method are in Additional file 1. Briefly, genomic DNA was extracted from 100 µl buffy coat containing blood leukocytes and platelets. For amplification of the Hp1 allele-specific 1757-bp sequence and Hp2 allele-specific 3481-bp sequence, the respective oligonucleotide primers A (5’-GAGGGGAGCTTGCCTTTCCATTG-3’) and B (5’-GAGATTTTTGAGCCCTGGCTGGT-3’) were used. For amplification of the other Hp2 allele-specific 349-bp sequence, the oligonucleotide primers C (5’-CCTGCCTCGTATTAACTGCACCAT-3’) and D (5’-CCGAGTGCTCCACATAGCCATGT-3’) were used [17]. Ethical approval did not extend to sequencing for point mutations in the Hp gene; accordingly this was not pursued.

### Statistics

Values are given as medians/means and IQR/SD. Correlations were analyzed using Spearman’s rank order test. Other comparisons were by Mann-Whitney U-test on paired or unpaired comparisons as indicated. A *p*-value of < 0.05 was considered statistically significant for all analyses. Coefficients of variation (CV%) were calculated as SD/mean x 100.

## Results

### Demographics, classification and clinical chemistry

Table 1 shows demographic data of the three groups of children, the 77 included for the allelic frequency, 66 of those for the paired higher/lower disease activity analyses and 42 healthy controls. The duration between paired visits in the 66 children with JIA was 1.5 ± 1.4 years (mean ± SD). Because there was no inclusion bias based on classification, the number of patients across classifications presumably reflects local relative incidences.

**Table 1:** Characteristics of 77 patients with juvenile idiopathic arthritis (JIA), and 42 healthy children in the study

	Total cohort, for allelic frequency	Participants for paired samples	Healthy controls
Participants, n (% females)	77 (61.0)	66 (62.1)	42 (30.9)
Age at sampling (Md, IQR)	10.3 (4.7–14.4)	10.1 (5.2–14.4)	7.7 (2.7–12.2)^c^
Age at onset (Md, IQR)	8.5 (3.2–12.4)	8.5 (3.2–12.6)	
**ILAR category** ^**a**^ **course type, n (%)**			
Oligoarticular^b^	38 (49.4)	32 (48.5)	
Polyarticular, RF-negative	16 (20.8)	14 (21.2)	
Polyarticular, RF-positive	3 (3.9)	3 (4.5)	
Enthesitis-related	10 (13.0)	7 (10.6)	
Psoriatic	5 (6.5)	5 (7.6)	
Systemic	1 (1.3)	1 (1.5)	
Undifferentiated	4 (5.2)	4 (6.1)	

*Md* Median, *IQR* inter-quartile range, *RF* rheumatoid factor

^a^According to the criteria established by the International League of Associations for Rheumatology (ILAR). The classification was established at inclusion and corrected for course type at follow-up

^b^persistent or extended

^c^Age at sampling in the healthy children compared to the total cohort of patients with JIA, p = N.S. Mann Whitney U test

Table 2 presents treatments and concentrations of liver related proteins in the 66 JIA children at the two disease activity states and applicable corresponding values for controls. The majority (*n* = 39, 59%) of the patients were included at onset of disease, before they had received any anti-inflammatory agents except for NSAIDs in some of them, in a higher disease activity state. The rest of the cohort (*n* = 27, 41%) were included when they were in a flare of the disease; only five of those had any kind of medical treatment at inclusion. Medications are presented at inclusion and at follow-up but not during the time in between. All APPs changed in the direction of healthy controls at lower disease activity, albeit total protein concentration was slightly, but significantly, higher than healthy controls.

**Table 2:** Clinical and laboratory characteristics of 66 patients with juvenile idiopathic arthritis (JIA), collected at two different study visits, at a higher and a lower disease activity state

	Children with JIA, *n* = 66		
**Disease activity**	Higher	Lower	*p*-value*
JADAS27 Md (IQR)	11.4 (6.8 – 17.1)	1.3 (0.1 – 2.8)^b^	< 0.001
**Medical treatment**			
No medical treatment	61	15	-
Methotrexate (MTX)	3	27	-
MTX + bDMARD	1	12	-
bDMARD		10	-
MTX + bDMARD + prednisolone		1	-
bDMARD and prednisolone		1	-
prednisolone	1		-
**Liver related proteins, Md (IQR)**			*p*-value*
Haptoglobin, g/L	1.5 (0.8 – 2.6)	0.7 (0.4 – 1.0)	< 0.001
Albumin, g/L	41 (37 – 43)	44 (42 – 46)	< 0.001
CRP, mg/L	2.2 (0.5 – 14.0)	0.4 (0.2 – 1.2)	< 0.001
Serum amyloid A, mg/L, (*n*=27)	1.7 (0.3 – 10.5)	0.4 (0.3 – 0.9)	< 0.001
Total protein, g/L^a^	74 (69 – 79)	77 (72 – 82)	< 0.05

*JADAS27* Juvenile Arthritis Disease Activity Score (0-57), *bDMARD* biological disease-modifying antirheumatic drugs, *Wilcoxon signed rank test. Reference values established by clinical chemistry unit were: Hp 0.1 – 1.8 g/L; Albumin 35-49 g/L; CRP <5 mg/L. ^a^Healthy participants were measured in this study as 70 (66 - 76). Md, Median; IQR, inter-quartile range. ^b^At the “Lower” activity visit, 32 patients (48%) met remission criteria of JADAS27 <1.0

### Western blot performance

**Fig. 1:**
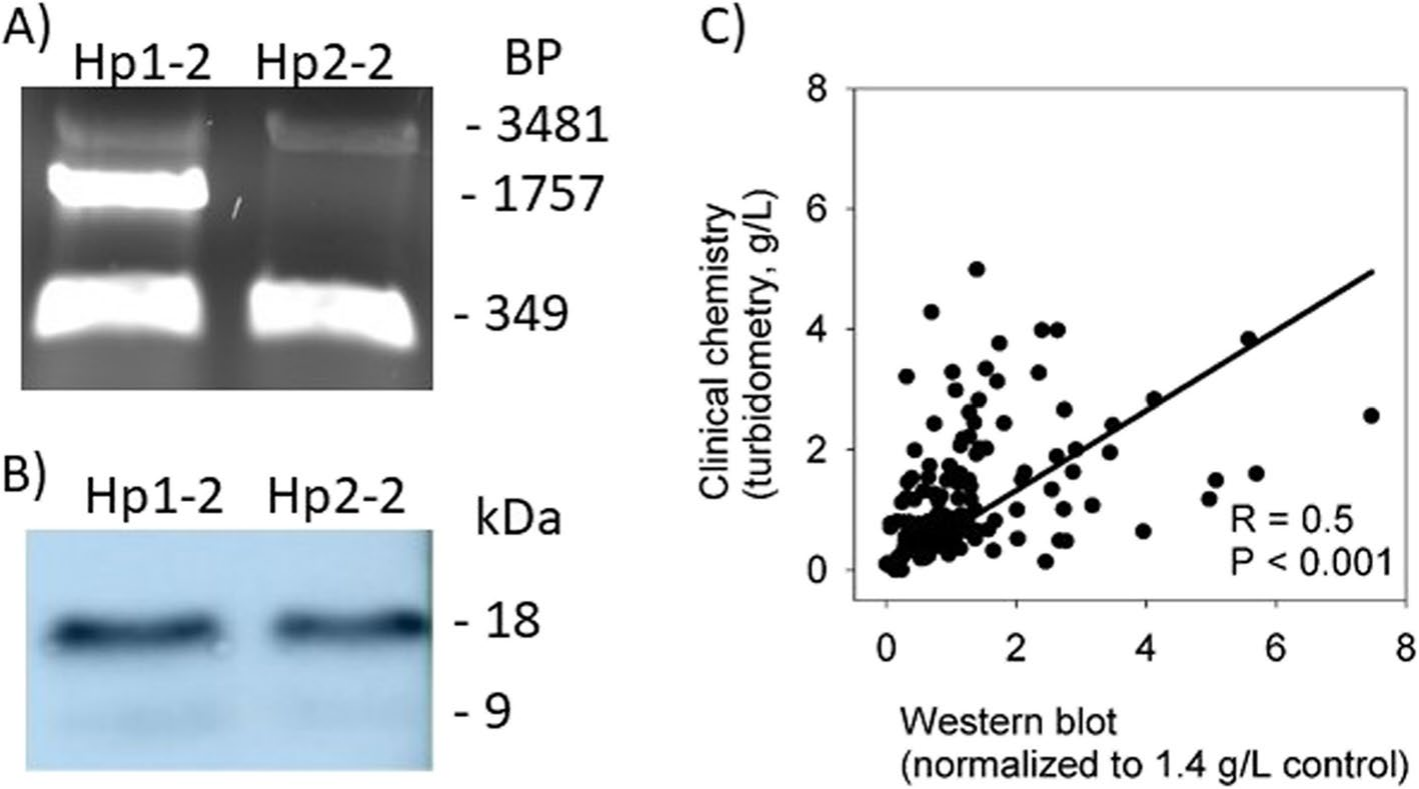
DNA and protein analyses of Hp alleles and comparison between Western blot and immunoturbidimetry.** A** Agarose gel displaying pooled results of A-B and C-D PCR reactions for DNA analysis. Hp1 allele is the middle 1757-bp band; Hp2 allele appears at both the top (3482-bp) and bottom (349-bp) bands. **B** Corresponding Western blot protein bands from same JIA patients. The α1 subunit is approximately ~ 9 kDa. The α2 variant is ~ 18 kDa. Note that the heterozygote blood sample had negligible level of α1 band and the α2 band was comparable to an Hp2 homozygote. **C** Correlation between Western blot densitometry and clinical chemistry immunoturbidimetry values. Combined Western blot densitometry values (α1 + α2) at higher versus lower disease activity (i.e., JADAS27 score) are plotted against corresponding values from validated immunoturbidimetry used in routine clinical chemistry. Western blot values were normalized against a reference plasma run on all gels, which was a heterozygote with Hp 1.4 g/L

Fig. 1 conveys assay performance considerations. Figure 1A shows DNA results for an Hp1-2 heterozygote and for comparison an Hp2-2 homozygote in which in both cases the α1 band was below signal to noise level to be considered positive in Western blot analysis (Fig. 1B). This isolated case exemplifies a possibility for some individuals to not have detectable Hp protein concentration of one allele despite possessing it in DNA. The DNA tests otherwise aligned with Western blot data. The sum of densitometry values for both Hp isoforms (α1 + α2) for each JIA patient for both visits was then plotted against Hp concentrations obtained from immunoturbidimetry to evaluate correlation between the two methods (Fig. 1C). Despite the semi-quantitative nature of Western blot and undefined binding properties of immunoturbidimetry for the various forms of Hp, the two methods correlated. Divergences from correlations relating to different antibodies and known Hp amino acid mutations were considered. Patients with most extreme divergences between assays differed at both visits with some individuals having consistently higher WB values than immunoturbidimetry and others having consistently lower WB values, consistent with differences due to antibodies and mutations. Other cases could be explained by also having values near the lower detection limit where accuracy suffers. One JIA patient was below WB detection limit with immunoturbidimetry giving 0.1 g/L at low activity visit; both Hp assays also gave low values at the high activity visit. Another patient was barely above WB detection whereas immunoturbidimetry was below detection limit; both Hp assays gave high values at the high activity visit. In one patient, WB value was 2-fold higher and IT was 10-fold lower at the lower activity visit. The two assays otherwise generally paralleled other.

### Hp allelic forms

Table 3 shows expression of the α1 and α2 Hp forms obtained by Western blot. The frequency of occurrence of the different forms was similar between JIA and healthy controls, albeit α1 bands were in most cases weaker than α2 (healthy and JIA), this explained by lower overall Hp concentrations in healthy controls.

**Table 3:** Percent Hp frequencies in plasma by Western blot in 77 children with juvenile idiopathic arthritis (JIA) and 37 healthy children

	Heterozygote	α1 Homozygote	α2 Homozygote
JIA (77)	67.5	3.9	28.6
Healthy (37)^a^	64.9	0	35.1

Hp: allele of haptoglobin; ^a^5 of the 42 subjects were ambiguous Hp2-2 or Hp1-2 due to low total Hp concentrations. These subjects could only be classified as likely Hp2 positives due to presence of Hp2 band at threshold of detection

### Hp at low and high disease activity

**Fig. 2:**
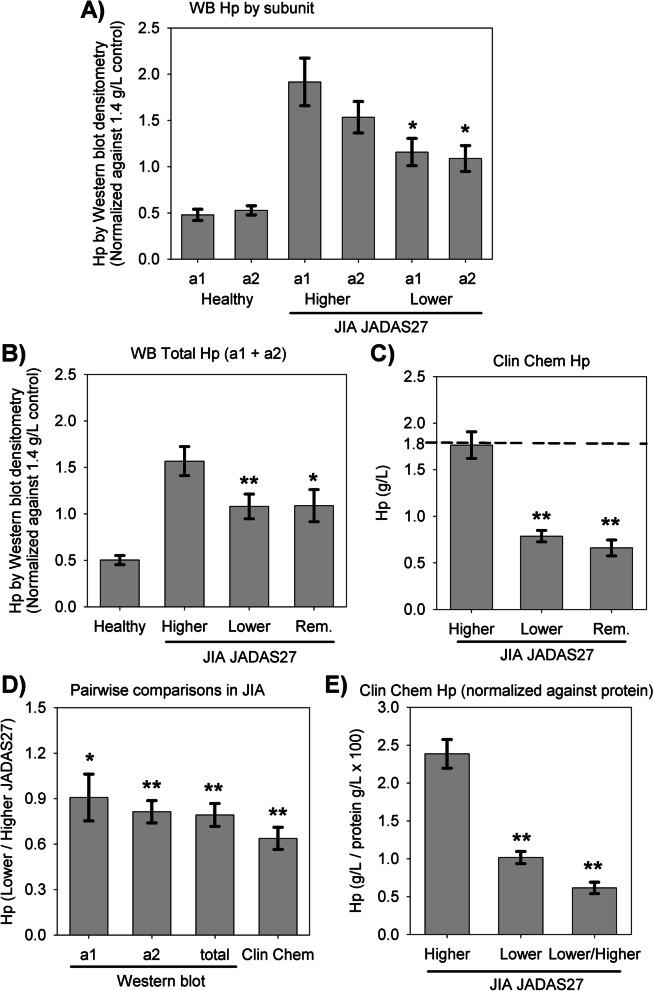
Post-treatment reduction in Hp detected by Western blot and immunoturbidimetry.** A** Densitometry values for Hp from Western Blot with α-chains measured separately. Each gel was run with the same reference “QC” sample from a healthy adult with 1.4 g/L Hp measured at Clinical Chemistry unit by immunoturbidimetry as well as JIA patient higher and lower JADAS27 samples run in parallel on same gel. For each resulting membrane, patient bands were normalized against the reference QC sample. That is, a patient α2 band with same densitometry value as reference QC would have a normalized α2 value of 1.0 (controls *n* = 39, higher and lower JADAS27 pairs *n* = 65 from JIA patients, unpaired statistics) **B** Densitometry values for sum α1 + α2 combined (controls *n* = 39, patients *n* = 65, JADAS27 < 1 (remission) *n* = 30, unpaired statistics) corresponding to total Hp measured by immunoturbidity at Clinical Chemistry unit. **C** Hp concentrations for same JIA patients measured by immunoturbidity at Clinical Chemistry unit. Dashed line indicates 1.8 g/L upper cutoff of reference range for 12–17 year olds (unpaired statistics). **D** Ratio of Hp values Lower/Higher Hp and significances of pairwise comparisons. **E** Clinical chemistry unit Hp concentrations by immunoturbidimetry normalized against total protein determination by Bradford method. This was done so as to compare to Western blot method, which loads lanes according to protein concentration. Statistical symbols are * *p* > 0.05 and ** *p* < 0.001 and describe higher vs. lower JADAS27 comparisons. JIA patients had higher Hp than healthy controls across all comparisons (*p* < 0.001). Significance symbols were not added for this type of comparison to avoid redundancy

Fig. 2 details different measures of Hp concentration. Figure 2A demonstrates that healthy children possess a densitometry of 0.5 relative the internal healthy adult reference. This was expected since adults are known to have somewhat higher Hp concentration than children. At higher individual disease activity defined by JADAS27 scores, both α1 and α2 densitometry values were higher than the reference. At lower disease activity, both bands declined significantly to the level of the reference, this remaining higher than healthy controls. The same was obtained comparing sum of α1 + α2 (Fig. 2B), which is methodologically more comparable to immunoturbidimetry in routine clinical chemistry that does not differentiate between the difference forms, which is shown in Fig. 2C. Isolating the 32 children in remission according to JADAS27, a score below 1.0, [20] as a separate group yielded essentially same results. The lower Hp concentration at lower disease activity was of comparable magnitude as obtained by Western blot. Hp was not measured by this method in the healthy children because there was reference data available from the clinical chemistry unit (dashed line at 1.8 g/L is upper cutoff). Although Hp concentration was higher when JADAS27 was higher, Hp concentration was below the clinical chemistry unit’s established upper cutoff. Hp mutations are associated with a- and hypo-haptoglobinemia. The lowest Hp concentration in the high activity visits was 0.2 g/L, occuring in only 3 patients (2 in the oligoarticular group; 1 in the polyarticular RF negative group). Lower cutoff of reference interval is typically 0.1 g/L.

Figure 2D shows pairwise comparisons by using fold differences between visits and indicates that individual Hp concentrations drop in the ballpark of 10 to 30% from higher to lower disease activity.

One methodological difference of Western blot compared to routine immunoturbidimetry is that total protein concentrations are measured for the purpose of equilibrating to same protein concentrations added to gel lanes. Due to the spread and differences in albumin and total protein concentrations (Table 2) at lower vs. higher JADAS27, the effect of normalizing for protein concentration as is done for Western blot was explored for the immunoturbidimetry data. Normalization for protein concentration did not have a dramatic effect on the magnitude of changes or *p*-values obtained (compare Fig. 2E C and 2D).

### Hp and other disease activity measures

Finally, correlations of APPs, ESR and JADAS27 score were explored (Table 4). Hp by immunoturbidimetry correlated better with JADAS27 than by Western blot, likely explainable by the quantitative design of immunoturbidimetry for clinical chemistry. Albumin also correlated with JADAS27, and being the only negative APP in this study, was tested to identify any improved correlation if used ratiometrically with Hp performed at the clinical chemistry unit (Hp(IT)/Alb). The correlation and *p*-value were unchanged. Fluctuating (e.g., oscillating) patterns of individual biomarkers would result in weaker correlations than without fluctuations, and would require substantial standard deviations about a mean concentration (i.e., high CV%). In the 27 patients of which all values were available at the higher disease activity visit, CV% was: Hp 74; CRP 183; SAA 225; albumin 7; ESR 107. Hence, fluctuating CRP and SAA in JIA are conceivable, whereas ESR and Hp cannot experience large amplitude fluctuations and albumin variability is too small to detect fluctuations (assays used to measure proteins typically have CV% of 5–10%).

**Table 4:** Spearman rank correlation analysis of liver derived APP concentrations and JADAS27 scores in juvenile idiopathic arthritis. ESR is included separately because it is only one component of JADAS27 scoring, which allows for comparison to Hp and other APPs.

	Hp(IT)	CRP	SAA	Albumin	Hp(IT)/Alb	JADAS27	ESR
**Hp(WB)**	0.489	0.442	0.165	-0.358	0.501	0.308	0.436
	< 0.001	< 0.001	0.2	< 0.001	< 0.001	< 0.001	< 0.001
	132	136	58	132	132	136	135
**Hp(IT)**		0.716	0.6	-0.532	0.994	0.592	0.613
		< 0.001	< 0.001	< 0.001	< 0.001	< 0.001	< 0.001
		132	54	132	132	132	132
**CRP**			0.814	-0.502	0.726	0.472	0.662
			< 0.001	< 0.001	< 0.001	< 0.001	< 0.001
			58	132	132	136	135
**SAA**				-0.28	0.594	0.496	0.629
				0.04	< 0.001	< 0.001	< 0.001
				54	54	58	57
**Albumin**					-0.601	-0.443	-0.525
					< 0.001	< 0.001	< 0.001
					132	132	132
**Hp(IT)/Alb**						0.608	0.626
						< 0.001	< 0.001
						132	132
**JADAS27**							0.485
							< 0.001
							139

Top numbers are Spearman rank order correlation coefficients; Middle numbers are *p* values; Bottom numbers are sample sizes and reflect all data available to make that paired comparison. *APP* acute phase proteins, *JADAS27* Juvenile Arthritis Disease Activity Score (0–57), *CRP* C-reactive protein, *ESR* erythrocyte sedimentation rate, *Hp* haptoglobin, *Hp(WB)* haptoglobin by Western blot, *Hp(IT)* haptoglobin by immunoturbidimetry, *SAA* serum amyloid A

In Additional file 2, CV% for Hp and JADAS27 are presented, all at the higher disease activity visit, reflected by JADAS27.

## Discussion

Our study showed that the frequency of occurrence of the allelic Hp isoforms was similar between JIA and healthy controls, supporting that children with JIA have similar possibility to produce pre-Hp2 (i.e. zonulin) in the context of intestinal permeability. At higher disease activity, Hp concentration correlated with other liver derived APPs. CRP correlation to JADAS27 was similar to other APPs.

At lower JADAS27 visits (i.e., during or after treatment), Hp, CRP and SAA were lower and albumin was higher. Hp half-life is about 2–4 days [23], although this is sharply reduced in the presence of substantial free hemoglobin, which is uncommon in JIA. ESR half-life presumably parallels decline in fibrinogen, which has a half-life of 3–5 days [23, 24] (i.e., similar to Hp). In adult rheumatoid arthritis, CRP half-life is 19 h [25]; as much as 3 days has been reported in other diseases [26]. In normal physiology, CRP concentration does not have any major cyclic patterns. However, under inflammatory conditions, oscillations in concentrations with a period of 6–7 days and amplitude of 2–20 fold have been reported in which troughs could drop below upper reference cutoff [27]. The present CV% data allows for the possibility that CRP and SAA markedly fluctuate in a time scale of several days and might explain some observations of normal CRP in presence of positive finds of other signs of inflammation. By inference, during high inflammatory activity roughly 1 in 7 blood samplings could be at a trough and another 1 in 7 at a peak. Such patterns have never been studied or demonstrated in JIA for any APP. This study identified three patients, traced to oligoarticular and polyarticular categories, with strong divergence in which strongly elevated ESR occurred (43–47 mm/h) in concomitance with normal CRP (1.5–4.2 mg/L); large temporal fluctuations in CRP might therefore exist in JIA. Another reason could be a difference in APPs related to JIA classification, although the present subgroups were too small to investigate with strong statistical power. The CV% find of large heterogeneity across classifications clearly warns that substantial sample sizes from each category would be needed to identify differences associated with disease category.

The failure of any of the liver proteins, having different half-lives, to correlate very strongly to JADAS27 score seemingly rules out half-life or periodicity as an explanation. This suggests that in some JIA patients IL-6 signaling cannot be very active during times of high disease activity. Another possibility is that the composite JADAS score does not reflect disease activity very well. This also implies that no permutation of alternative measures of liver derived APPs (e.g., Hp allelic forms, ratiometric, etc.) is likely to show any stronger correlation with the other JADAS parameters than ESR or CRP.

Earlier research found moderate rather than strong correlation between CRP and ESR in JIA, but composite JADAS scorings using either CRP or ESR were deemed equally valid for calculating JADAS because the two calculations strongly correlated [28]. This is because ESR and CRP have limited contribution to JADAS scoring. ESR for example, below 20 mm/h does not influence the total count. Number of active joints greatly influences total count irrespective affected joint sizes, which some experts propose be considered [29].

Higher concentrations than controls of Hp and other liver-related proteins even in a lower disease activity state establishes guidance on how gut permeability models for zonulin in JIA pathogenesis should be envisioned. Circulating Hp concentration may be chronically elevated in some patients even at a JADAS27 score of < 1, which could be the case in bile, enabling a mode by which the gut epithelium experiences supra-physiological exposure to zonulin or other permeability mediators. Hp in JIA pathogenesis, as through effects on the GI-tract, could be heterogeneous, further suggesting heterogeneity in responsiveness to anti-IL6 therapy.

## Strengths and limitations

This is the first study to explore different forms of Hp at different disease activities in JIA and to relate to other disease activity indicators. This data can be used for power analysis to plan future studies. Some JIA classifications were only rarely encountered, so less confidence can be placed in data stratified by category, albeit the large heterogeneities were not confined to any one category. One strength was that the many APPs including isoforms of Hp, were analyzed and evaluated in the same cohort. Because Hp concentration can be obtained by capillary blood tap, a practical role for Hp in clinical chemistry for assessment of disease activity in JIA patients should be studied.

## Conclusion

Plasma Hp1 and Hp2 concentrations are both higher in JIA than healthy children, both decline at lower disease activity, but remain higher than healthy controls, even at JADAS27 < 1. Frequency of occurrence of the mature proteins does not differ from healthy controls. Circulating Hp concentrations largely parallel other APPs and ESR, but none of these measures correlate very strongly to JADAS27 score. The reason for this is not related to half-life or which forms of APPs (e.g., Hp1 vs. Hp2) are measured. Chronically higher Hp concentration than healthy children appears to be a feature of JIA. This should be studied in more detail to address questions regarding APP effects on GI physiology (e.g., hyper-permeability leading to immune activation) and anti-IL6 effectiveness or dosing regimens. Hp is a stable protein with a half-life close to ESR that, unlike ESR, is possible to analyze from capillary samples.

## Supplementary Information

**Additional file 1.**

**Additional file 2.**

## Data Availability

The datasets used and/or analysed during the current study are available from the corresponding author on reasonable request.

## Abbreviations

APP: Acute phase protein
CRP: C-reactive protein
CV%: Coefficient of variation (stdev/mean x 100)
ESR: erythrocyte sedimentation rate
GI: Gastro-intestinal
Hp: Haptoglobin
IL: Interleukin
ILAR: International League of Associations for Rheumatism criteria
IQR: Interquartile range
JADAS27: Juvenile arthritis disease activity score 27 joints
JIA: Juvenile idiopathic arthritis
Md: Median
NSAIDs: Non-steroidal anti-inflammatory drugs
PCR: Polymerase chain reaction
SAA: Serum amyloid A
SD: Standard deviation
WB: Western blot
