# Supplementary material for: Haptoglobin in Juvenile Idiopathic Arthritis
Source: Pediatr Rheumatol Online J. 2022 Dec 15;20:117. doi: 10.1186/s12969-022-00777-5 (PMC9753416; doi:10.1186/s12969-022-00777-5)
Supplement: Supplementary file 1 — Additional file 1. [file 12969_2022_777_MOESM1_ESM.docx]

***Additional file 1 – Detailed description of Western blot and PCR.***

***Western blot***

Herein, Hp1 refers to the shorter ancestral form with the corresponding peptide possessing one α subunit that gives rise to the 9 kDa subunit (α1). Hp2 refers to the longer allelic form possessing a duplication-fusion (effectively two tandem α subunits) that results in an ~18 kDa subunit (α2). Plasma protein concentrations were measured by Bradford method. Four sample buffer formulations were tested (Tris-SDS pH 6.8 or 8.3 with or without 12.5 mM EDTA at 1x). The pH 8.3 with EDTA formulation performed best, and was used. Plasma was diluted 1:300 (needed to avoid overloading lanes and resolve bands) in H_2_O and added to sample buffer with further addition of β-ME (final 5% v/v) added prior to boiling for 30 min. Precast 12% Mini-PROTEAN® TGXTM 10-well gels were used for PAGE-SDS (BioRad, Cat: 4561043). Lanes were loaded with 1 µg protein. Each gel had one molecular weight marker lane and another for a reference sample (healthy plasma from heterozygote with BSA-FITC). Primary antibody was mouse monoclonal (clone E-9, IgG2b, Santa Cruz Biotech, Cat# sc-374208, dilution 1:200). Detection was enhanced chemiluminescence using horse-radish preoxidase (HRP)-tagged secondaries screened for mouse IgG2b binding. Densitometry was performed on both α-chains separately; ImageJ software was used to demarcate blank or Hp band regions of interest and then obtain mean signal intensity of the pixels within [1]. Lower limit of detection (LLOD) was defined as 3 standard deviations above mean of background, which was considered suitable for a positive score for phenotyping. Lower limit of quantification (LLOQ) was used for threshold to quantify α chain concentrations, defined as 10 standard deviations above background. Relative concentrations were calculated as ratio to the reference sample bands present on all membranes. The reference sample had the following concentrations: albumin (43 g/L), Hp (1.4 g/L by routine clinical chemistry immunoturbidimetry), protein (63 g/L). The sum of the α1 and α2 densitometries was used for “total Hp”. The stoichiometry between α and β subunits was assumed to be 1:1 (i.e., the densitometric sum of mature α subunits equals total mature Hp monomers). In other words, a sum equal to that of the reference sample was assigned a value of 1.0; a sum half of the reference was assigned a value of 0.5, etc.

***DNA analysis of Hp alleles by PCR***

Genomic DNA was extracted from 100 µl buffy coat containing blood leukocytes and platelets by DNeasy® Blood & Tissue Kit (Cat No./ID: 69506, Qiagen). For amplification of the Hp1 allele-specific 1757-bp sequence and Hp2 allele-specific 3481-bp sequence, the respective oligonucleotide primers A (5’-GAGGGGAGCTTGCCTTTCCATTG-3’) and B (5’-GAGATTTTTGAGCCCTGGCTGGT-3’) were used. For amplification of the other Hp2 allele-specific 349-bp sequence, the oligonucleotide primers C (5’-CCTGCCTCGTATTAACTGCACCAT-3’) and D (5’-CCGAGTGCTCCACATAGCCATGT-3’) were used [2]. Two separate reactions using primers A-B or C-D were performed with Platinum™ Taq DNA Polymerase (Cat 10966018, Invitrogen) as follows: initial denaturation 95˚C 2 min, denaturation 95˚C 30 sec, annealing 65˚C 30 sec, extension 72˚C 3 min with 30 cycles and a final extension at 72˚C 10 min. The two resulting mixtures for each subject were pooled and reaction products (3 in total) separated on 0.7% agarose gel. Ethidium bromide fluorescence was used for imaging. Ethical approval did not extend to sequencing for point mutations in the Hp gene; accordingly this was not pursued.

1. Schneider CA, Rasband WS, Eliceiri KW. NIH Image to ImageJ: 25 years of image analysis. Nature Methods. 2012;9(7):671-5. doi: 10.1038/nmeth.2089.

2. Levy AP, Asleh R, Blum S, Levy NS, Miller-Lotan R, Kalet-Litman S, et al. Haptoglobin: Basic and Clinical Aspects | Antioxidants & Redox Signaling. 2010.
