# Supplementary material for: Haptoglobin in Juvenile Idiopathic Arthritis
Source: Pediatr Rheumatol Online J. 2022 Dec 15;20:117. doi: 10.1186/s12969-022-00777-5 (PMC9753416; doi:10.1186/s12969-022-00777-5)
Supplement: Supplementary file 2 — Additional file 2. [file 12969_2022_777_MOESM2_ESM.docx]

**Supplementary Table 1.** CV% for Haptoglobin and JADAS27 are presented in 66 children with juvenile idiopathic arthritis, all at the higher disease activity visit reflected by JADAS27.

|  | Haptoglobin g/L | | JADAS27^a^ |  |
| --- | --- | --- | --- | --- |
|  | Mean (SD) | CV% | Mean (SD) | CV% |
| **ILAR category^b^ course type (n)** |  |  |  |  |
| Oligoarticular^c^ (32) | 1.7 (1.1) | 61.9 | 8.6 (5.6)** | 64.9 |
| Polyarticular, RF-negative (14) | 1.6 (1.4) | 85.0 | 16.9 (9.2)** | 54.2 |
| Polyarticular, RF-positive (3) | 2.3 (0.6) | 27.3 | 21.7 (5.1)** | 23.7 |
| Enthesitis-related (7) | 2.1 (1.2) | 54.8 | 16.3 (4.5)** | 27.8 |
| Psoriatic (5) | 1.0 (0.7) | 68.0 | 14.0 (4.1) | 29.7 |
| Systemic (1) | 4.3 (N/A) | N/A | 13.2 (N/A) | N/A |
| Undifferentiated (4) | 1.8 (1.4) | 73.1 | 9.1 (4.3) | 47.7 |

^a^JADAS27= juvenile arthritis disease activity score 27.

^b^According to the criteria established by the International League of Associations for Rheumatology (ILAR).

^c^persistent or extended.

N/A, not applicable for n=1.

** Oligoarticular group was lower than the other 3 classes at p>0.01. Kruskal-Wallis One Way Analysis of Variance on Ranks was used. There were no other significant differences.
